# The Impact of DAXX, HJURP and CENPA Expression in Uveal Melanoma Carcinogenesis and Associations with Clinicopathological Parameters

**DOI:** 10.3390/biomedicines12081772

**Published:** 2024-08-06

**Authors:** Alexandros Pergaris, Georgia Levidou, Georgios Mandrakis, Maria-Ioanna Christodoulou, Michail V. Karamouzis, Jerzy Klijanienko, Stamatios Theocharis

**Affiliations:** 1 First Department of Pathology, Medical School, National and Kapodistrian University of Athens, 11527 Athens, Greece; alexperg@yahoo.com (A.P.); giormandr@biol.uoa.gr (G.M.); 2 Department of Pathology, Paracelsus Medical University, 90419 Nuremberg, Germany; georgia.levidou@klinikum-nuernberg.de; 3 Tumor Immunology and Biomarkers Laboratory, Basic and Translational Cancer Research Center, Department of Life Sciences, European University Cyprus, Nicosia 2404, Cyprus; mar.christodoulou@euc.ac.cy; 4 Molecular Oncology Unit, Department of Biological Chemistry, Medical School, National and Kapodistrian University of Athens, 11527 Athens, Greece; mkaramouz@med.uoa.gr; 5 Department of Pathology, Curie Institute, 75005 Paris, France; jerzy.klijanienko@curie.fr

**Keywords:** uveal melanomas, cancer, DAXX, HJURP, CENPA, biomarkers, prognosis

## Abstract

Uveal melanomas (UMs) represent rare malignant tumors associated with grim prognosis for the majority of patients. DAXX (Death Domain-Associated Protein), HJURP (Holliday Junction Recognition Protein) and CENPA (Centromere Protein A) proteins are implicated in epigenetic mechanisms, now in the spotlight of cancer research to better understand the molecular background of tumorigenesis. Herein, we investigated their expression in UM tissues using immunohistochemistry and explored possible correlations with a multitude of clinicopathological and survival parameters. The Cancer Genome Atlas Program (TCGA) was used for the investigation of their mRNA levels in UM cases. Nuclear DAXX expression correlated with an advanced T-stage (*p* = 0.004), while cytoplasmic expression marginally with decreased disease-free survival (DFS) (*p* = 0.084). HJURP nuclear positivity also correlated with advanced T-status (*p* = 0.054), chromosome 3 loss (*p* = 0.042) and increased tumor size (*p* = 0.03). More importantly, both nuclear and cytoplasmic HJURP immunopositivity correlated with decreased overall survival (OS) (*p* = 0.011 and 0.072, respectively) and worse DFS (*p* = 0.071 and 0.019, respectively). Lastly, nuclear CENPA overexpression was correlated with presence of irido-corneal angle involvement (*p* = 0.015) and loss of chromosome 3 (*p* = 0.041). Nuclear and cytoplasmic CENPA immunopositivity associated with decreased OS (*p* = 0.028) and DFS (*p* = 0.018), respectively. *HJURP* and *CENPA* mRNA overexpression exhibited strong association with tumor epithelioid histology and was linked to worse prognosis. Our results show the compounding role of DAXX, HJURP and CENPA in UM carcinogenesis, designating them as potential biomarkers for assessing prognosis and possible targets for novel therapeutic interventions.

## 1. Introduction

Intraocular melanomas comprise a rare malignancy, with 3490 cases reported in the United States in 2023 [1]. The eye represents the second most common site where melanoma arises [2]. Uveal melanoma (UM), in particular, constitutes the most common type of intraocular melanomas, amounting to an estimated 85% of cases [3]. Although their incidence reaches only six cases per million [3], they are accompanied by a remarkably unfavorable prognosis. Metastatic disease at the time of diagnosis is the rule [4,5] and patients’ relative survival is equal to 79 and 66% at 5 and 10 years, respectively [6]. Moreover, disease recurrence is frequent, even years after initial diagnosis [4,5]. Fair skin color, light-colored eyes and dysplastic nevus syndrome comprise predisposing factors for the appearance of UM [7]. As far as patients’ survival is concerned, adverse prognostic factors include tumor thickness, epithelioid cell morphology, increased mitotic activity and chromosome 3 monosomy [8,9,10,11,12] It is, therefore, apparent that novel biomarkers are needed to aid in timely diagnosis as well as in the development of novel, personalized and effective therapeutic interventions.

Epigenetic mechanisms have gathered the interest of the scientific community, as they are considered to hold a key role in the process of carcinogenesis. They are implicated in a wide variety of cellular processes that drive neoplasia, including the regulation of expression of genes controlling cell cycle progression, proliferation and apoptosis [13,14,15]. Among the countless proteins participating in the regulation of genome expression, DAXX (Death Domain-Associated Protein), HJURP (Holliday Junction Recognition Protein) and CENPA (Centromere Protein A) represent three biomolecules implicated in many physiologic processes as well as the pathogenesis of a plethora of tumors [16,17,18,19,20,21,22,23,24,25].

DAXX represents a specific H3.3 histone chaperone and participates in a variety of complex molecular mechanisms implicated in oncogenesis. For example, DAXX is part of the TGFβ-induced apoptotic mechanism via JNK activation and the FAS-DAXX-ASK1-MAP2K pro-apoptotic pathway [17,26]. Aberrations in DAXX expression have been well-documented in a plethora of tumors [16]. HJURP interacts with CENPA, acting as its chaperone, and plays a crucial role in securing chromosome stability. P53 has been observed to bind to the promoters of CENPA and HJURP, resulting in the downregulation of their expression. P53 mutations are accompanied by HJURP and CENPA upregulation, triggering their oncogenic properties [27].

Our current research focused on exploring the expression of DAXX, HJURP and CENPA in human UM tissues and their possible associations with a broad range of patients’ clinicopathological characteristics, aiming at the designation of the aforementioned proteins as valuable prognostic biomarkers, as well as possible targets for future, personalized treatment regimens.

## 2. Materials and Methods

### 2.1. Patients

This is a study of archival histopathological material from 49 patients with UM diagnosed in 2007–2008 at Institut Curie, for whom medical records were available. Data on long-term survival were available for 47 out of 49 patients. All patients underwent a surgical enucleation without any radiotherapy or chemotherapy before surgical procedure. In this study, well known prognostic parameters for UM such as tumor size, intra- or extrascleral extension, histological grade, cell type, mitotic activity and presence of metastasis were recorded. Moreover, three additional parameters that most strongly affect the visual acuity, namely tumor location in the posterior pole, retinal detachment and vitreous hemorrhage were also taken into consideration. Data regarding chromosome 3 loss and the gain of chromosome 8 were available in a small subset of our cohort. The histological cell type was evaluated by hematoxylin and eosin (H&E) staining according to the modified Callender classification system. Tumor size was defined as the largest basal diameter (in mm). Mitotic activity was assessed on ×400 in 40 fields using H&E staining. The presence of tumor infiltrating lymphocytes (TILs) or peritumoral lymphocytes was evaluated in a H&E staining assessing the whole tumor area. The clinicopathological characteristics of the cases included in this study are presented in Table 1.

### 2.2. Immunohistochemistry

We carried out immunohistochemistry using standard procedures in formalin-fixed paraffin-embedded (FFPE) UM tissue sections. The sections were stained with antibodies against human DAXX (clone ab239806, AbCam, Cambridge, UK, at dilution 1:50), HJURP (clone ab100800, AbCam, at dilution 1:5000) and CENPA (clone Ab217622, AbCam, at dilution 1:200). Antigen retrieval was performed at pH 6. The Envision visualization system (Dako, Agilent, Santa Clara, CA, USA) was used according to the manufacturer’s instruction. DAB (3,3-diaminobenzidine) was used as a chromogen, and hematoxylin as a counterstain. Gastric adenocarcinoma tissues were used as positive controls for DAXX antibody. Tissues from our previous works were used as positive controls for HJURP and CENPA antibodies [28]. As a negative control, the omitted primary antibody and substitution with an irrelevant antiserum was used.

The evaluation of immunohistochemistry (IHC) was conducted independently by two pathologists (A.P. and S.T.) blinded to patients’ information and clinicopathological characteristics. Nuclear and cytoplasmic immunoreactivity were evaluated separately. The percentage of tumor cells exhibiting nuclear and cytoplasmic staining for DAXX, HJURP and CENPA to the total number of tumor cells within each section was calculated. Staining intensity was evaluated using 4 categories: 0 (no reaction), 1 (mild reaction), 2 (moderate reaction) and 3 (intense reaction). H-score calculation comprised of multiplying the semiquantitative staining intensity score (score 0 to 3) by the percentage of positive cells, ranging, therefore, between 0 and 300.

### 2.3. Statistical Analysis

Statistical analysis was performed by an MSc biostatistician (G.L.). The association between the immunohistochemical expression of DAXX, HJURP and CENPA with the recorded clinicopathological parameters was examined using non-parametric tests with correction for multiple comparisons (Kruskal–Wallis ANOVA, Mann–Whitney U test, and Spearman’s correlation coefficient), as appropriate. Survival analysis for overall survival (OS) and disease-free survival (DFS) was performed using Kaplan–Meier survival curves and the differences between the curves were compared with log-rank test. Due to the limited sample size, a multivariate survival analysis was not performed. A *p*-value of <0.05 was considered statistically significant. A *p*-value of >0.05 but lower of <0.10 was considered of marginal significance. The analysis was performed with the statistical package STATA 11.0/SE for Windows.

### 2.4. The Cancer Genome Atlas Program (TCGA)

We explored TCGA in order to extract data regarding the mRNA expression of *DAXX*, *HJURP* and *CENPA* in UM tissues. The aforementioned mRNA levels were examined in 80 UM cases. We proceeded to correlate the results with clinicopathological parameters available, including tumor histology and patients’ prognosis. The analysis was performed with the software tool UALCAN (The University of ALabama at Birmingham CANcer data analysis Portal) (https://ualcan.path.uab.edu/).

## 3. Results

### 3.1. DAXX IHC Expression and Association with Clinicopathological Parameters

**Figure 1:**
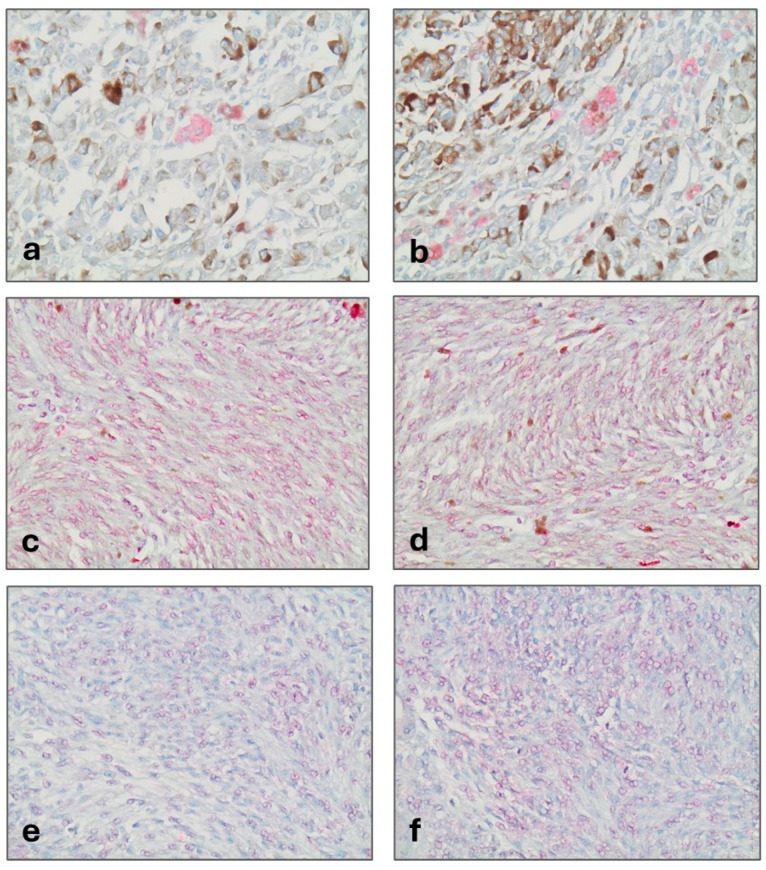
DAXX (**a**,**b**), HJURP (**c**,**d**) and CENPA (**e**,**f**) exhibited both nuclear and cytoplasmic staining (×400).

DAXX immunoreactivity was both nuclear and cytoplasmic (Figure 1). Nuclear immunopositivity was observed in 13 (27.6%) and cytoplasmic in 19 cases (40.4%). The median DAXX nuclear and cytoplasmic H-score was 0 (a range of 0–40 for nuclear and of 0–210 for cytoplasmic). A total of 14 cases (29.8%) displayed only cytoplasmic immunoexpression, 8 (17%) only nuclear, 5 (10.6%) both nuclear and cytoplasmic reactivity and 20 (42.6%) were completely negative. There was not any significant association between nuclear and cytoplasmic DAXX expression (Spearman’s correlation coefficient, R = 0.07, *p*= 0.6314).

**Figure 2:**
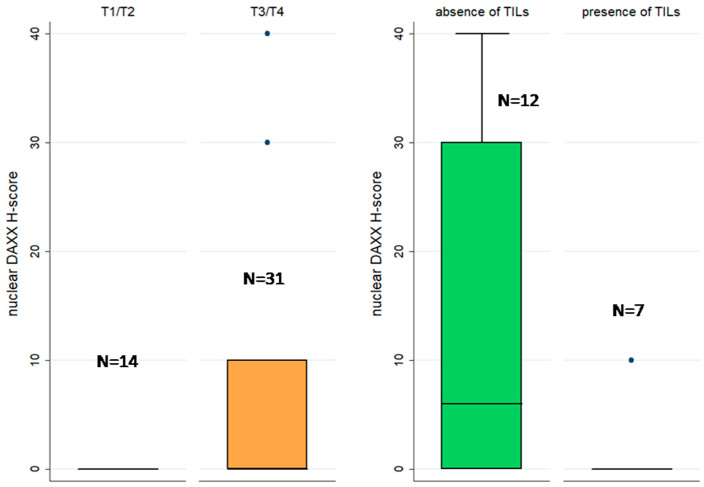
Schematic representation of the associations between nuclear DAXX H-score and T-status (**left**, Mann–Whitney U test, *p* = 0.005, N = 45) and TILs (**right**, Mann–Whitney U test, *p* = 0.039, N = 19).

Cases with retinal detachment and advanced T-status showed a higher nuclear DAXX H-score (Mann–Whitney U test, *p* < 0.001 for retinal detachment and *p* = 0.005 for T-status, Figure 2). In the same context, the presence of nuclear DAXX expression was correlated with advanced T-stage (Fischer’s exact test, *p* = 0.004). Interestingly, an increased DAXX H-score was correlated with the absence of tumor-infiltrating lymphocytes (TILS) (Mann–Whitney U test, *p* = 0.039, Figure 2). A respective association with peritumoral lymphocytes was not observed. Moreover, cases with a positive nuclear DAXX staining displayed a higher number of mitoses, compared to the negative ones, a relationship which, however, was of marginal significance (Mann–Whitney U test, *p* = 0.059). On the other hand, the absence of cytoplasmic DAXX immunoexpression was correlated with decreased number of mitoses (Mann–Whitney U test, *p* = 0.034).

**Figure 3:**
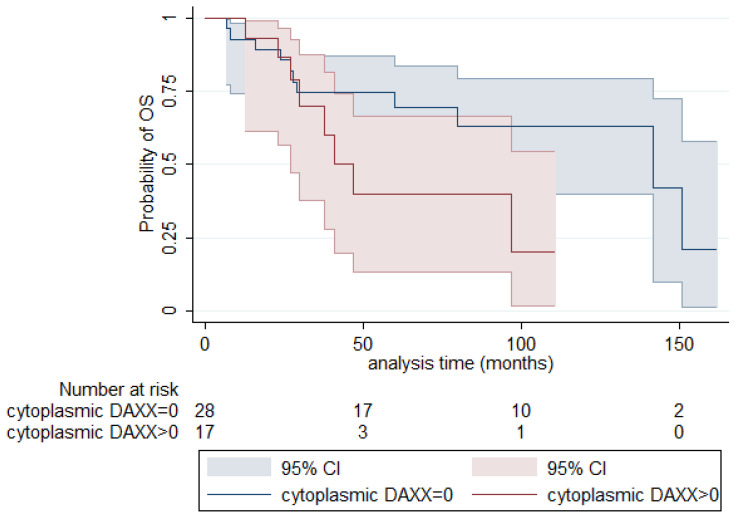
Relationship of cytoplasmic DAXX with DFS (log-rank test, *p* = 0.084).

The presence of cytoplasmic expression was marginally correlated with decreased DFS, the positive cases showing a median DFS of 29.5 months compared to 61 months for the negative cases (log-rank test, *p* = 0.084, Figure 3). The respective correlation with OS was not significant. Moreover, nuclear DAXX expression did not show any significant association with either OS or DFS.

The rest associations of nuclear and cytoplasmic DAXX immunoreactivity with the parameters presented in Table 1 were not significant (*p* > 0.10).

### 3.2. HJURP IHC Expression and Association with Clinicopathological Parameters

HJURP expression was also nuclear and cytoplasmic (Figure 1). Nuclear immunopositivity was observed in 18 (36.7%) and cytoplasmic in 28 (57.1%) of cases. A total of 8 cases (16.3%) displayed only nuclear immunoreactivity, 18 (36.7%) only cytoplasmic, 10 (20.5%) showed both nuclear and cytoplasmic expression and 13 (26.5%) were completely negative.

**Figure 4:**
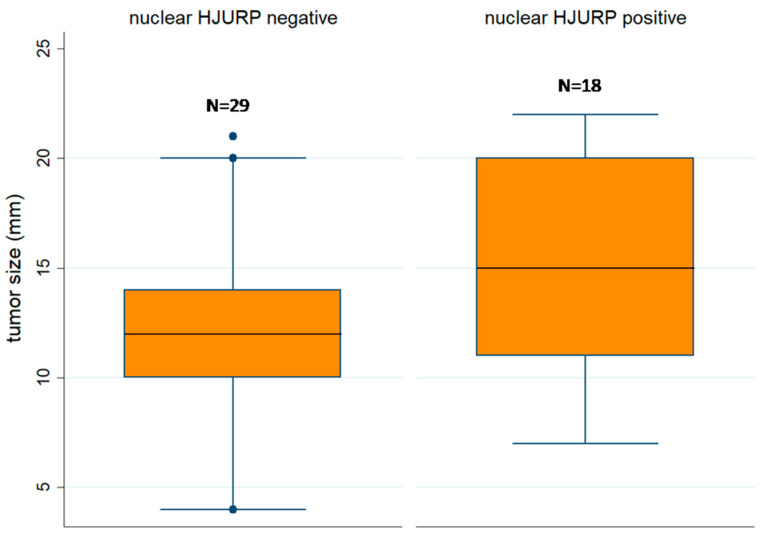
Schematic representation of the associations between nuclear HJURP positivity and tumor size (Mann–Whitney U test, *p* = 0.030, N = 47).

All cases with HJURP nuclear immunopositivity displayed chromosome 3 loss, whereas only half of the cases which were HJURP negative had a chromosome 3 loss (Fischer’s exact test, *p* = 0.042). Moreover, nuclear HJURP positivity was correlated with advanced T-status (Fischer’s exact test, *p*= 0.054, I versus II/III/IV, 44,4% versus 17.2%) and increased tumor size (Mann–Whitney U test, *p* = 0.030, Figure 4). Cytoplasmic HJURP H-score was higher in cases with ciliary body involvement (Mann–Whitney U test, *p* = 0.021).

**Figure 5:**
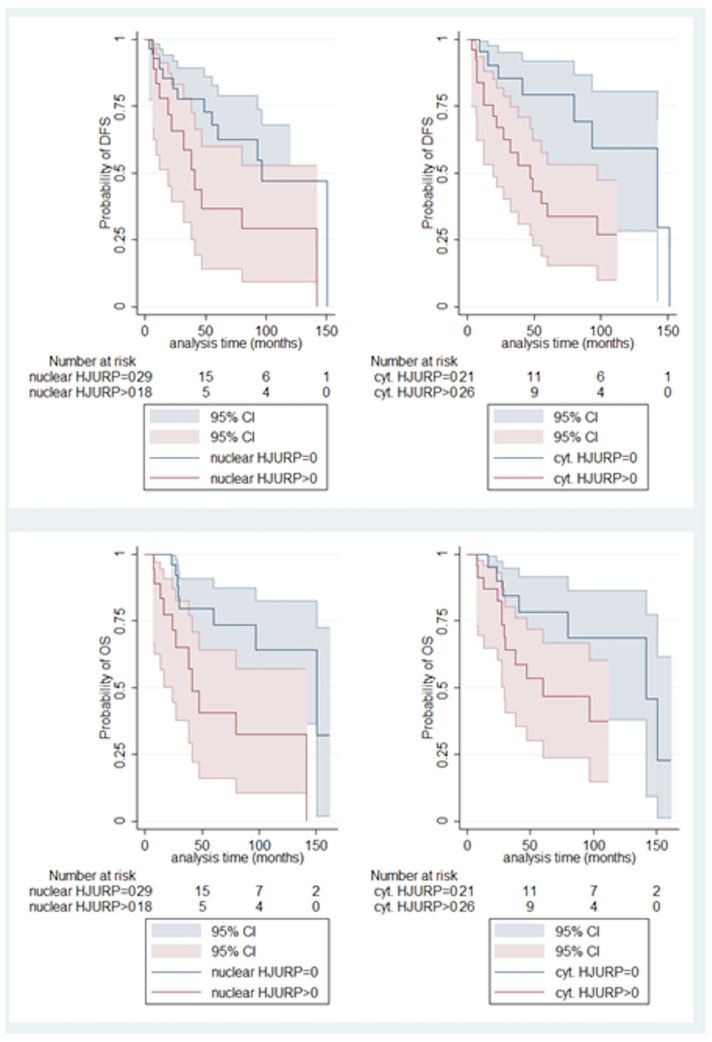
Relationships of nuclear HJURP with OS (**upper left**) and DFS (**upper right**) as well as of cytoplasmic HJURP with OS (**lower left**) and DFS (**lower right**).

Both nuclear and cytoplasmic HJURP immunopositivity were correlated with decreased OS (log-rank test, *p* = 0.011 for nuclear, *p* = 0.072 for cytoplasmic, Figure 5), the latter association being of marginal significance. Accordingly, both nuclear and cytoplasmic HJURP immunopositivity were correlated with worse DFS (log-rank test, *p*= 0.071 for nuclear, *p* = 0.019 for cytoplasmic Figure 5), the former association being of borderline significance.

**Figure 6:**
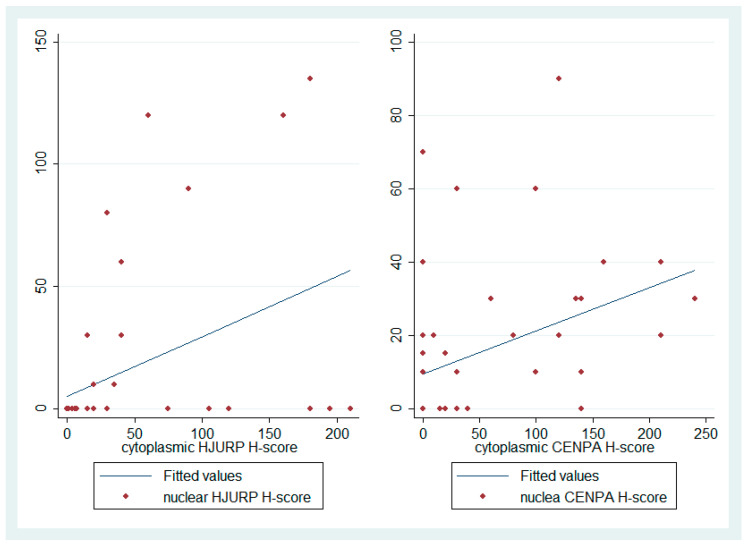
Schematic representation of the association between nuclear and cytoplasmic HJURP and CENPA H-score.

Moreover, there was a positive correlation between nuclear and cytoplasmic H-score (Spearman’s correlation coefficient, N= 49, R = 0.48, *p* < 0.001, Figure 6).

The rest associations of nuclear and cytoplasmic HJURP immunoreactivity with the parameters presented in Table 1, as well as with the presence of intratumoral (TILs) and peritumoral lymphocytes were not significant (*p* > 0.10).

### 3.3. CENPA IHC Expression and Association with Clinicopathological Parameters

CENPA expression in UMs was both nuclear and cytoplasmic (Figure 1). Nuclear immunopositivity was observed in 25 cases (52.1%) and cytoplasmic in 27 cases (56.3%). A total of 10 cases (20.8%) displayed only cytoplasmic immunoreactivity, 8 (16.7%) only nuclear, 17 (35.4%) showed both a nuclear and cytoplasmic expression and 13 (27.1%) were completely negative. There was a positive correlation between nuclear and cytoplasmic CENPA H-score (Spearman’s correlation coefficient, N = 49, R = 0.41, *p* < 0.001, Figure 6).

Increased nuclear CENPA H-score was correlated with the presence of irido-corneal angle involvement (Mann–Whitney U test, *p* = 0.015) and the loss of chromosome 3 (Mann–Whitney U test, *p* = 0.041). The same applied to cytoplasmic CENPA H-score, which seemed to be higher in cases with loss of chromosome 3 (Mann–Whitney U test, *p* = 0.078), a relationship, however, which was of marginal significance. Moreover, there was a tendency of frequently higher cytoplasmic CENPA H-score in cases with advanced T-status (Fischer’s exact test, *p* = 0.096, 37.5% versus 13.6%), but this correlation achieved only borderline significance.

**Figure 7:**
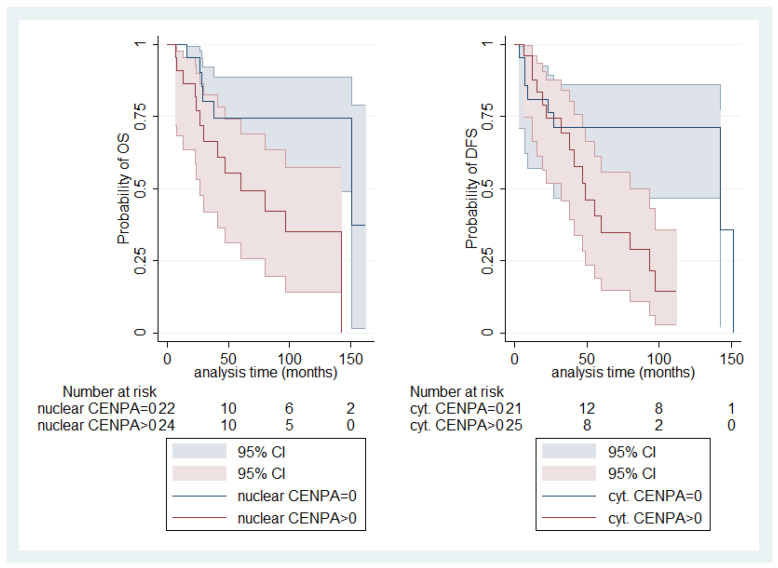
Relationship of nuclear CENPA positivity with OS (**left**) and cytoplasmic CENPA positivity with DFS (**right**).

Interestingly, nuclear CENPA immunopositivity was correlated with decreased OS (log-rank test, *p* = 0.028, Figure 7) and cytoplasmic CENPA immunopositivity with decreased DFS (log-rank test, *p* = 0.018, Figure 7). The respective correlation of nuclear CENPA with DFS and cytoplasmic CENPA with OS was not significant.

The rest associations of nuclear and cytoplasmic CENPA immunoreactivity with the parameters presented in Table 1, as well as with the presence of intratumoral (TILs) and peritumoral lymphocytes were not significant (*p* > 0.10).

### 3.4. TCGA Analysis

From the data analysis of the TCGA data, specifically TCGA-UM consortium that includes 80 patients in total, using web available software tools (UALCAN), some conclusions were drawn for HJURP, DAXX and CENPA gene expression, especially regarding associations with UM histology and patients’ prognosis.

**Figure 8:**
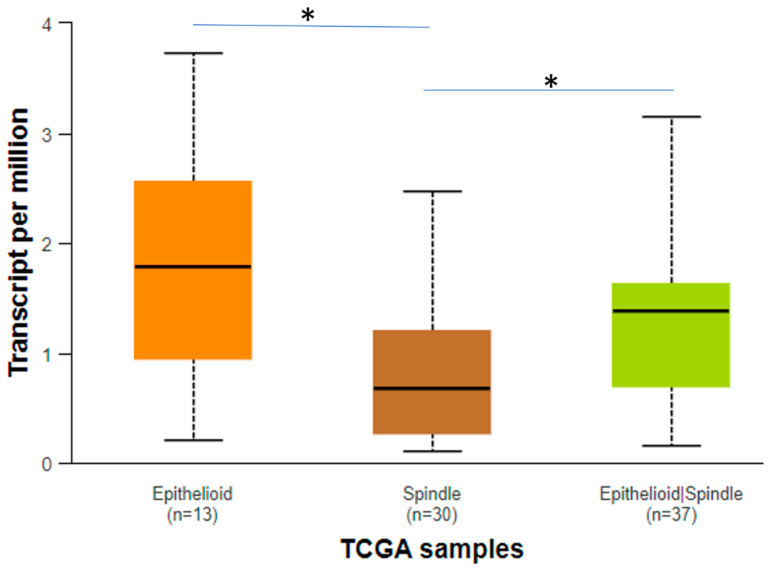
Expression of *CENPA* in different histological types. The statistically important comparisons are shown with a straight line and an asterisk (*). This asterisk means that the *p* value is less than 0.05.

**Figure 9:**
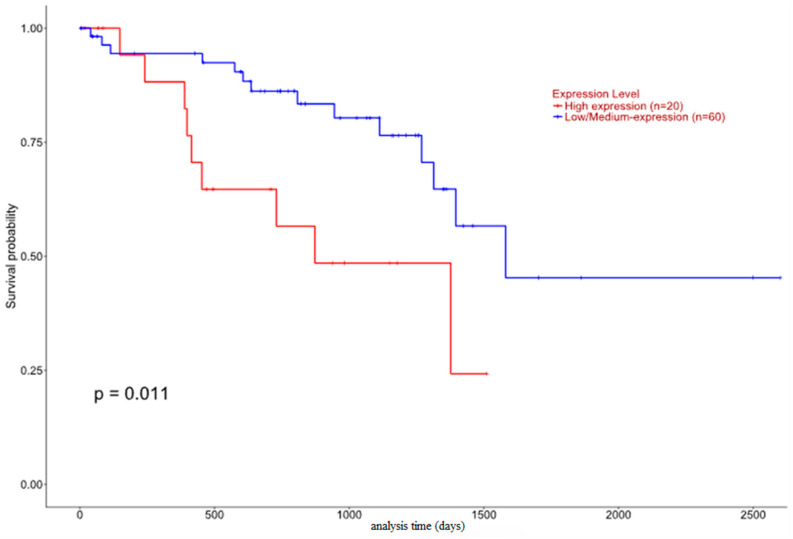
*CENPA* expression and patients’ survival.

Regarding CENPA, it is evident that it is significantly downregulated in the spindle histological type compared to the epithelioid and epithelioid/spindle histological types (Figure 8). Furthermore, the survival analysis revealed that the patient group that upregulates CENPA has worse prognosis compared to the low expression group (*p* = 0.01) (Figure 9).

**Figure 10:**
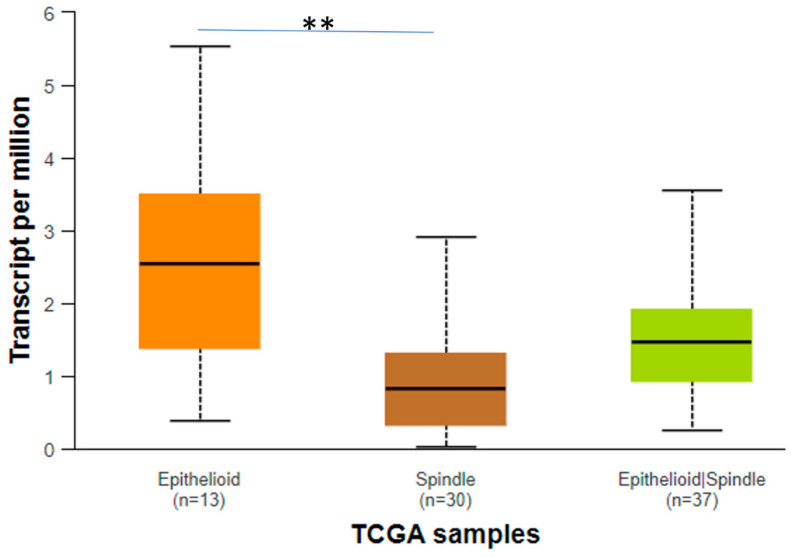
Expression of *HJURP* in different histological types. The statistically important comparisons are shown with a straight line and a double asterisk (**). The two asterisks mean that the *p* value is less than 0.01.

**Figure 11:**
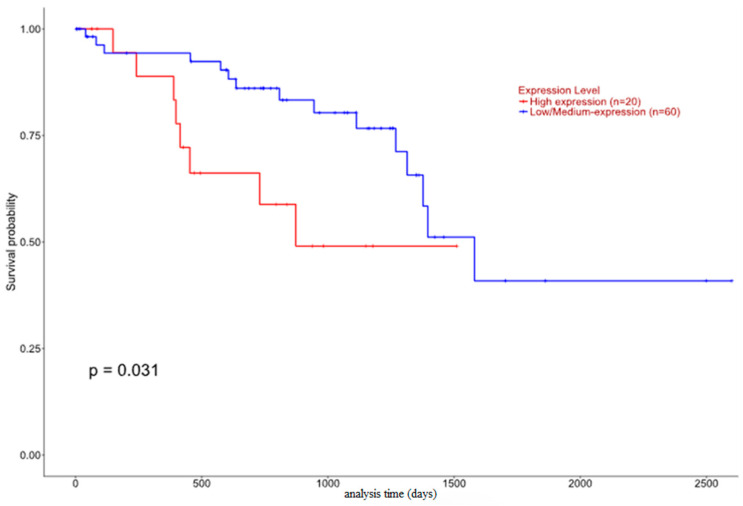
*HJURP* expression and patients’ survival.

As far as *HJURP* analysis is concerned, it is observed that the epithelioid histological type has a significantly increased expression compared to the spindle histological type (Figure 10). Moreover, the Kaplan–Meier graph demonstrates a higher survival probability to the high expression group compared to the low expression patient group, even though the survival rate drops sharper in the patient group that upregulates *HJURP* (*p* = 0.031) (Figure 11).

**Figure 12:**
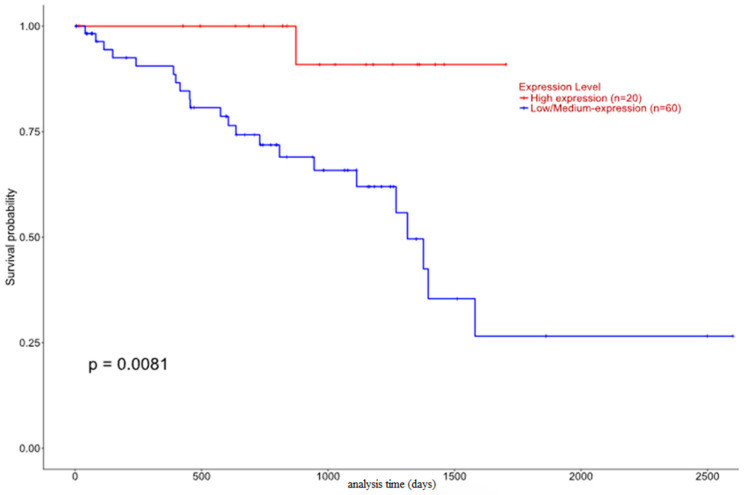
*DAXX* expression and patients’ survival.

While no statistically important data had been collected from the histological type expression analysis for *DAXX*, the survival analysis revealed a different pattern. It becomes apparent that *DAXX* downregulation is associated with decreased survival probability, whereas the patient group that exhibits increased *DAXX* mRNA expression has a better prognosis (Figure 12).

## 4. Discussion

UMs comprise a rare but serious disease, often accompanied by a grim prognosis and often a fatal outcome [6]. The development of novel therapeutic options and the accurate assessment of disease prognosis requires focusing on the molecular mechanisms behind UM tumorigenesis.

The epigenetic mechanisms shaping the complex landscape of protein expression have been in the epicenter of anti-cancer research for decades. Complicated molecular pathways contribute to the development of neoplasia by determining the silencing or overexpression of genes, and unveiling their role in each individual tumor type remains of utmost importance. Among the biomolecules implicated in cancer epigenetics, DAXX, HJURP and CENPA represent three proteins heavily impacting oncogenesis in a variety of tumors. In this context, DAXX overexpression is associated with ovarian [29] and prostate carcinogenesis [19] but appears to hold tumor-suppressing properties as well [16]. Similarly, both tumor-promoting and tumor-suppressing properties are attributed to HJURP overexpression, depending on the type of tissue [16]. Lastly, CENPA overexpression is reported as present in many tumors [23].

Our present study attempts to shed light on the implication of DAXX, HJURP and CENPA in the carcinogenesis of UMs and associate their immunohistochemical expression in tumor tissues with patients’ clinicopathological parameters and prognosis.

In our study, immunohistochemical DAXX was associated with traditional negative prognostic parameters in UM, namely advanced T disease stage and the number of mitoses [12,30,31,32,33]. The association between DAXX expression and tumor stage is in keeping with the results of other investigations in prostate cancer [30] and chondrosarcomas [31]. Similarly, cytoplasmic DAXX expression was marginally associated with decreased patients’ DFS, and nuclear DAXX overexpression was correlated with the absence of TILs, an observation which is reportedly linked to decreased patients’ OS [12,34,35]. On the other hand, the data we extracted from TCGA also indicated that increased DAXX mRNA expression was linked to better patients’ prognosis. This discrepancy with the results of immunohistochemical analysis may indicate that DAXX mRNA and protein expression as well as subcellular protein location (in the nucleus or the cytoplasm) might influence UM pathogenesis in different ways. It is apparent that the role of DAXX expression in UM pathogenesis is complex and needs to be further clarified.

Interestingly, immunohistochemical HJURP expression in our study seemed to be associated with many parameters, implicated in worse patients’ prognosis in UM. Firstly, HJURP nuclear immunopositivity was correlated with increased tumor size, and cytoplasmic HJURP overexpression was linked to ciliary body involvement, all factors heralding a grimmer patients’ prognosis when present [12]. Importantly, nuclear HJURP positivity was associated with advanced T-status, in accordance with previous investigations in other tumors, such as in lung [36,37], kidney [38], prostate carcinoma [39] and cholangiocarcinoma [40]. In the same context, we observed an association between both nuclear and cytoplasmic HJURP immunopositivity with decreased patients’ OS. This finding is in alignment with similar observations in a variety of human malignancies, including tumors of the ovaries [21,41], endometrium [42], oral cavity [43], kidney [38,44], breast [45,46], lung [36,37,47,48], liver [49,50] and pancreas [51], as well as in hematologic malignancies [52], gliomas [53], cholangiocarcinomas [40] and cutaneous melanomas [54]. We also underlined an association between nuclear and cytoplasmic HJURP immunopositivity and decreased DFS, a phenomenon reported in many studies in other tumors [21,37,44,45,50]. In contrast, however, to our immunohistochemical results in TCGA samples higher HJURP expression was associated with a higher survival probability.

Moreover, in our investigation, increased nuclear CENPA expression was associated with loss of chromosome 3 and appears to be associated with decreased OS. However, the correlation with chromosome 3 loss should be considered preliminary, since this information was only available in a small subset of our cohort. In the same context, cytoplasmic CENPA immunopositivity was associated with decreased DFS. Additionally, data from TCGA also supported that *CENPA* mRNA upregulation was linked to worse prognosis. Similar associations between *CENPA* expression and worse patients’ outcome have been described in a multitude of studies regarding other tumors as well [55,56,57,58].

The present investigation is the first study analyzing concomitantly the expression of HJURP, CENPA and DAXX in UMs. It has, however, some limitations that need to be taken into consideration. For example, the limited sample size did not allow us to perform a multivariate survival analysis to explore the possibility of cofactors in the relationships of these molecules with OS or DFS. However, it should be mentioned that the results of the survival analysis in our investigation recapitulate many of the traditional parameters that have been proposed as important determinants of the clinical outcome in UMs, supporting the validity of our statistical analysis and denoting that our cohort is representative. Given the retrospective nature of our investigation, further prospective investigations exploring also mechanistic pathways are warranted in order to explore potential causal relationships.

## 5. Conclusions

In an attempt to explore the role of three molecules implicated in key epigenetic procedures, namely DAXX, HJURP and CENPA, in UMs, we observed that the expression of all aforementioned proteins plays a compounding role in UM carcinogenesis and is further associated with worse patients’ prognosis. Our findings are in keeping with a plethora of studies in the literature that also attribute to those three proteins a negative influence in the tumorigenesis of many tumor types. More studies are needed, however, in order to establish a solid and causal association between the expression of DAXX, HJURP and CENPA and clinicopathological parameters and designate these proteins as useful prognostic biomarkers as well as targets for the development of novel, specialized therapeutic interventions.

## Figures and Tables

**Table 1:** Clinicopathological characteristics of UM patients included in this study (n = 49).

Parameter	Median	Range
Age	63	32–90 years
Number of mitoses per 40 HPFs	3	0–25
Tumor size	13	range 4–22 mm
Tumor thickness	9	range 1–16 mm
	**Number**	**%**
Gender		
Male	19	39%
Female	30	61%
Posterior pole involvement	31	63%
Ciliary body involvement	15	31%
Iris involvement	2	4%
Irido-corneal angle involvement	2	4%
Presence of retinal detachment	12	24.5%
Presence of vitreous hemorrhage	5	10%
Intrasclera involvement	40	81.6%
Extrasclera involvement	4	8%
Histological cell type		
Epithelioid cell	11	22.5%
Mixed cell	25	51%
Spindle cell	13	26.5%
Loss of chromosome 3	14	70%
Gain 8q	7	63.4%
Presence of metastasis	11	31.4%
T-category (AJCC)		
Τ1	1	2.1%
Τ2	14	29.8%
Τ3	19	40.4%
Τ4	13	27.7%
Event		
Death of disease	19, within 7–151 months	38.76%
Censored	28, follow-up 1–162 months	57.14%

## Data Availability

The original contributions presented in the study are included in the article, further inquiries can be directed to the corresponding author.
